# Assessing vertical transmission of SARS-CoV-2 in high-rise apartments via a joint epidemiologic and modeling investigation

**DOI:** 10.3389/fpubh.2025.1694554

**Published:** 2025-12-17

**Authors:** Samuel Z. Chong, Chin Chun Ooi, Muhammad Ismail Bin Abdul Malek, Zhengwei Ge, Derrick Low, Chang Wei Kang, Calvin J. Chiew, Sae-Rom Chae, Yee Leong Teoh, Kelvin Bryan Tan

**Affiliations:** 1 Ministry of Health, Singapore, Singapore; 2 Institute for High Performance Computing, Agency for Science, Technology and Research, Singapore, Singapore; 3 National Centre for Infectious Diseases, Singapore, Singapore; 4 Communicable Diseases Agency, Singapore, Singapore; 5 Saw Swee Hock School of Public Health, National University of Singapore, Singapore, Singapore; 6 Duke-NUS Graduate Medical School, National University of Singapore, Singapore, Singapore; 7 Lee Kong Chian School of Medicine, Nanyang Technological University, Singapore, Singapore

**Keywords:** SARS-CoV-2, epidemiologic investigation, computational fluid dynamics, vertical transmission, aerosol transmission, Urban Health, high rise apartment

## Abstract

High-rise apartments (HRAs) present a complex environment with multiple routes of infection, especially for respiratory pathogens like SARS-CoV-2. With HRAs serving as the dominant housing modality in many dense urban regions worldwide, particularly in dense Asian cities such as Singapore, where high-rise living is normative, understanding transmission within such settings is essential for both global and region-specific public health preparedness. In this study, we assessed transmission risks and potential routes of transmission within HRAs based on observed severe acute respiratory syndrome coronavirus 2 (SARS-CoV-2) clusters in Singapore. We analyzed SARS-CoV-2 incidence in HRAs subjected to government-mandated mass screenings to evaluate the transmission risk associated with various relative positions within an HRA and found significantly elevated risk of transmission for residents living within the same vertical stack as a potential index case. A computational fluid dynamics (CFD) model was further developed for an HRA with the highest vertical transmission risk to elucidate potential routes of aerosol transmission. Interestingly, the epidemiological analysis indicated an increased risk of infection for residents living within two levels above an infected case, correlating with CFD observations that aerosolized particles can move vertically up the stack and remain at elevated concentrations in the two levels above a potential index case. The analysis and modeling provide additional insights into alternative vertical transmission within HRAs, distinct from prior studies that have primarily hypothesized transmission via drainage stacks. Nonetheless, factors such as wind direction and individual unit configurations are shown in CFD to have a significant influence on the potential spread of aerosolized particles in such settings, highlighting the need for additional in-depth investigation. This study further demonstrates the importance of joint epidemiology and numerical modeling to better understand different potential mechanisms of particle spread, especially in the HRA setting.

## Introduction

Over the course of the severe acute respiratory syndrome coronavirus 2 (SARS-CoV-2) pandemic, significant scientific progress has been made in the characterization and understanding of airborne transmission of infectious respiratory diseases (1–3). Through rigorous analysis of infection incidents, a consensus on the possibility of airborne transmission across different scenarios has been gradually developed. SARS-CoV-2 is now known to spread primarily through respiratory droplets and aerosols released when an infected person coughs, sneezes, talks, or breathes, particularly in enclosed or poorly ventilated settings. Transmission may also occur via contact with contaminated surfaces followed by mucosal inoculation, including fecal-oral transmission, especially as the virus can persist on surfaces for hours to days, depending on environmental conditions. Infection then occurs when the SARS-CoV-2 virus successfully binds to the angiotensin-converting enzyme 2 receptor, with the serine protease TMPRSS2 facilitating viral entry and replication within the respiratory epithelium (4–6). In the past few years, multiple studies have examined the associated risk levels across different settings and conducted quantitative assessments of the effectiveness of various mitigation measures, including mask-wearing, use of various air purification devices, and the importance of effective ventilation systems within buildings (7–11). Many research papers in the current SARS-CoV-2 literature have reported the possibility and/or mechanism of airborne transmission across diverse indoor scenarios spanning different social settings, especially because relative physical proximity and reduced fresh air intake (in air-conditioned settings) can exacerbate the risk of infection (12–14). Recent meta-analyses further underscore that most SARS-CoV-2 transmission occurs via aerosols in indoor environments, with minimal spread observed outdoors (15). In particular, high-risk indoor activities that generate abundant aerosols, such as singing or intense exercise, are associated with the greatest transmission rates, underscoring the critical importance of ventilation in curbing airborne spread (16). Furthermore, these indoor social settings can be easily targeted for intervention, via implementation of social distancing measures or by improving fresh air intake (17).

On the contrary, there is a relative lack of studies on transmission within the residential setting, despite measures such as home quarantines or lockdowns being implemented over the course of the pandemic (18). More specifically, high-rise apartments (HRAs), by virtue of their density and complex structural interactions, may be of particular concern for transmission of infectious diseases, especially as they may be the dominant residence type in many dense urban metropolises (19). These settings present unique vulnerabilities that may exacerbate the spread of pathogens, particularly in cities such as Singapore, where HRAs predominate, and deserve more in-depth studies to enhance awareness of potential routes of transmission and motivate corresponding directed mitigation strategies.

Earlier seminal research on SARS-CoV-1 has shown the possibility of transmission in an HRA setting (20). Similarly, transmission across vertical stacks (i.e., the column of units stacked directly above one another in the HRA) has been hypothesized in observed clusters during the SARS-CoV-2 pandemic, with a few initial reports of potentially related cases in apartment buildings spanning multiple countries, including China, Korea, and Canada (21–24). While these individual reports highlight a single cluster in a single apartment building, together they provide empirical evidence that aerosolized viral particles may indeed travel along the vertical axis of HRAs and cause infection across households. Interestingly, many of these SARS-CoV-2 studies have centered around the possibility of transmission via the bathroom (especially via the drainage ducts in the bathrooms), with little discussion of other possibilities thus far (21, 22, 25–28).

On 20 May 2021, the first cluster of SARS-CoV-2 cases in an HRA was identified in Singapore. As Singapore was still in a containment phase at the time, this prompted a compulsory screening exercise comprised of a polymerase chain reaction (PCR) test for SARS-CoV-2 for all residents staying in the HRA, which commenced on 21 May 2021. Over the course of the pandemic, multiple clusters in other HRAs were identified, and similar exercises were carried out. Comprehensive lists of individuals residing within these HRAs, and their relevant health history, were collected as part of these exercises. Hence, in this study, we utilized this unique and comprehensive dataset on SARS-CoV-2 cases in HRAs in Singapore, which underwent government-mandated mass screenings, to systematically assess the risk and characteristics of SARS-CoV-2 transmission across all reported HRA clusters during this period, thereby providing quantitative insights into the spatial dependence of risk of infection, including statistically significant evidence of vertical transmission.

Based on the epidemiological data, an HRA with the highest number of unique unit transmissions within a single vertical stack was selected for modeling with computational fluid dynamics (CFD) using the same methodology as employed in multiple prior studies on possible SARS-CoV-2 transmission (14, 29–31). Given that epidemiological results are derived from all observed cases across multiple buildings, this single set of CFD simulation results is more useful as a means of deriving mechanistic insights into alternate possible transmission processes (in addition to the more commonly studied drainage-related route), as opposed to generalizable, quantitative probabilities of infection. Through this model, it was observed that airborne vertical stack transmission consistent with the observed epidemiology for this particular local built environment can occur under an appropriate combination of prevailing winds and unit configuration (determined based on the meteorological conditions observed during the cluster outbreak). On the contrary, horizontal, same-level transmission was not observed under our set of (albeit limited) simulation scenarios, suggesting that other non-airborne transmission routes may be the potential cause of the increased infection risk observed in the epidemiology. Taken together, this study highlights the value and importance of a joint epidemiologic and modeling study for understanding airborne transmission of infectious diseases, especially with regard to understanding various potential transmission routes in the context of complex HRAs.

## Methods

### Epidemiology

Between 1st May and 20th September 2021, Singapore experienced relatively low levels of SARS-CoV-2 transmission in the community; this preceded the infection wave driven by the Delta variant. During this period, 18 HRAs were identified as SARS-CoV-2 clusters and were subjected to government-mandated mass testing exercises under the Infectious Diseases Act (IDA). Resident lists obtained during these exercises were used to identify all individuals residing in the 18 HRAs, and the demographic and household information gathered was deemed accurate and complete. Resident lists were matched against SARS-CoV-2 testing data in the COVID-19 Test Repository (CTR) using unique identification numbers collected during the mass testing exercises to verify SARS-CoV-2 positivity across the study period. There were no issues with matching, as all resident identifiers were complete and validated at the point of data collection. All confirmed COVID-19 infections in Singapore are recorded in the CTR and tagged to the same unique identifiers, enabling complete and accurate linkage between resident records and testing outcomes. In total, 8,909 residents were included in the study, among whom 265 tested positive for SARS-CoV-2 during the study period. Of these infections, 54 had no prior exposure to HRA infections in the preceding 7 days. They were excluded from the counts of secondary infections, but were included as potential index cases for other residents of the HRA.

With low incidence of cases and significant social measures such as safe distancing and masking in place during this period, there was reduced possibility of infection exposure from other sources in the community. Increased cleaning standards for these HRAs, such as higher-frequency cleaning and sanitization of high-contact-point areas, including central refuse chute, lift surfaces, handrails, and letterboxes, also reduced the possibility of fomite transmission.

**Figure 1:**
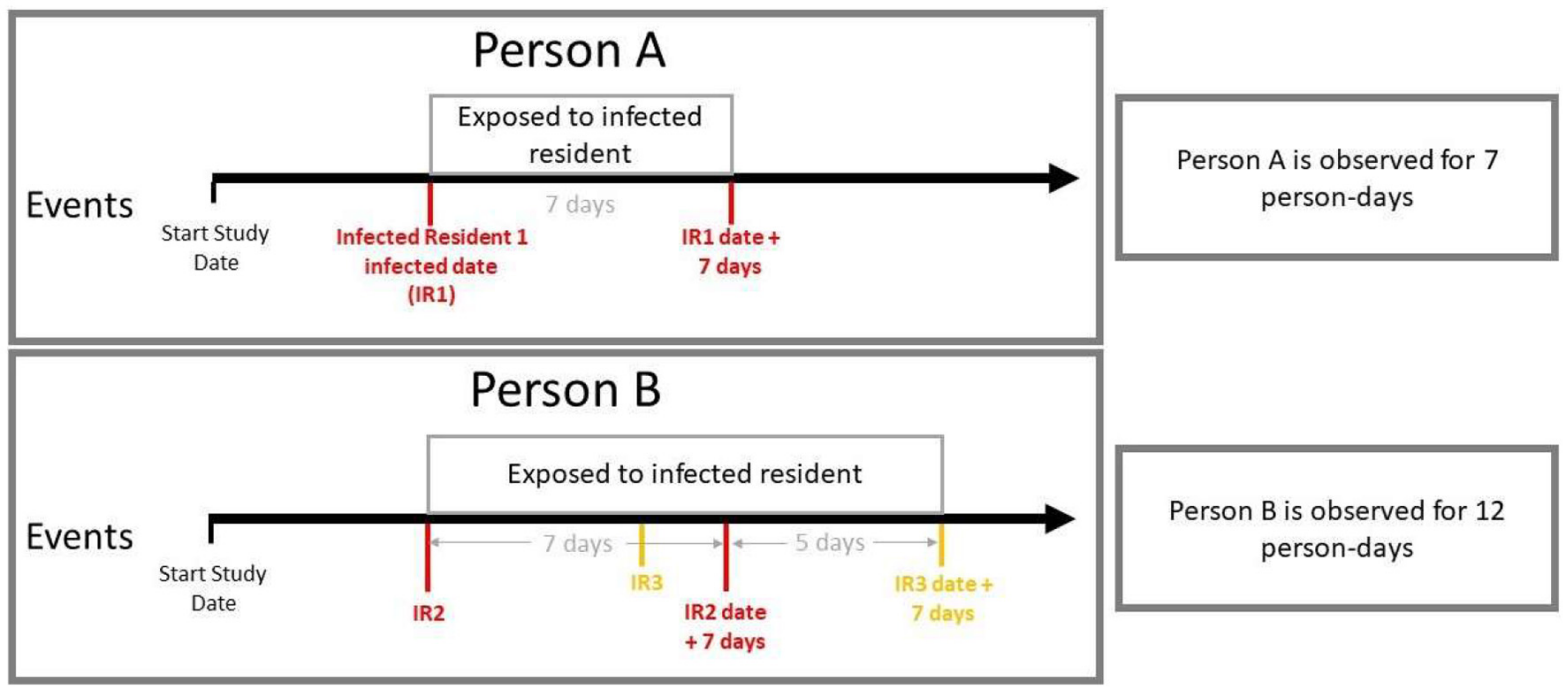
A schematic of how person-days are calculated for exposed individuals. We present two example timelines for two different individuals and detail the number of person-days they are at risk of transmission.

Analyses for all 18 HRAs across this period were set up on a person-day basis using methods previously described by Bar-On et al., adjusted for the covariates of age, sex, race, residency status, and vaccination status (32). As illustrated in Figure 1, person-day observation begins when a SARS-CoV-2-positive case is identified, with no other cases detected in the same HRA during the preceding 7 days. Subsequently, everyone within the same HRA is observed daily, and any new infection is considered a possible transmission from the index cases within the preceding 7 days, consistent with previous epidemiological studies (33, 34). These new cases would then be considered as a potential index case for the following seven person-days. Logistic regression was then used to estimate the odds of SARS-CoV-2 infection within 7 days among potentially vulnerable individuals living in the same household, level, and vertical stack as the index cases.

**Figure 2:**
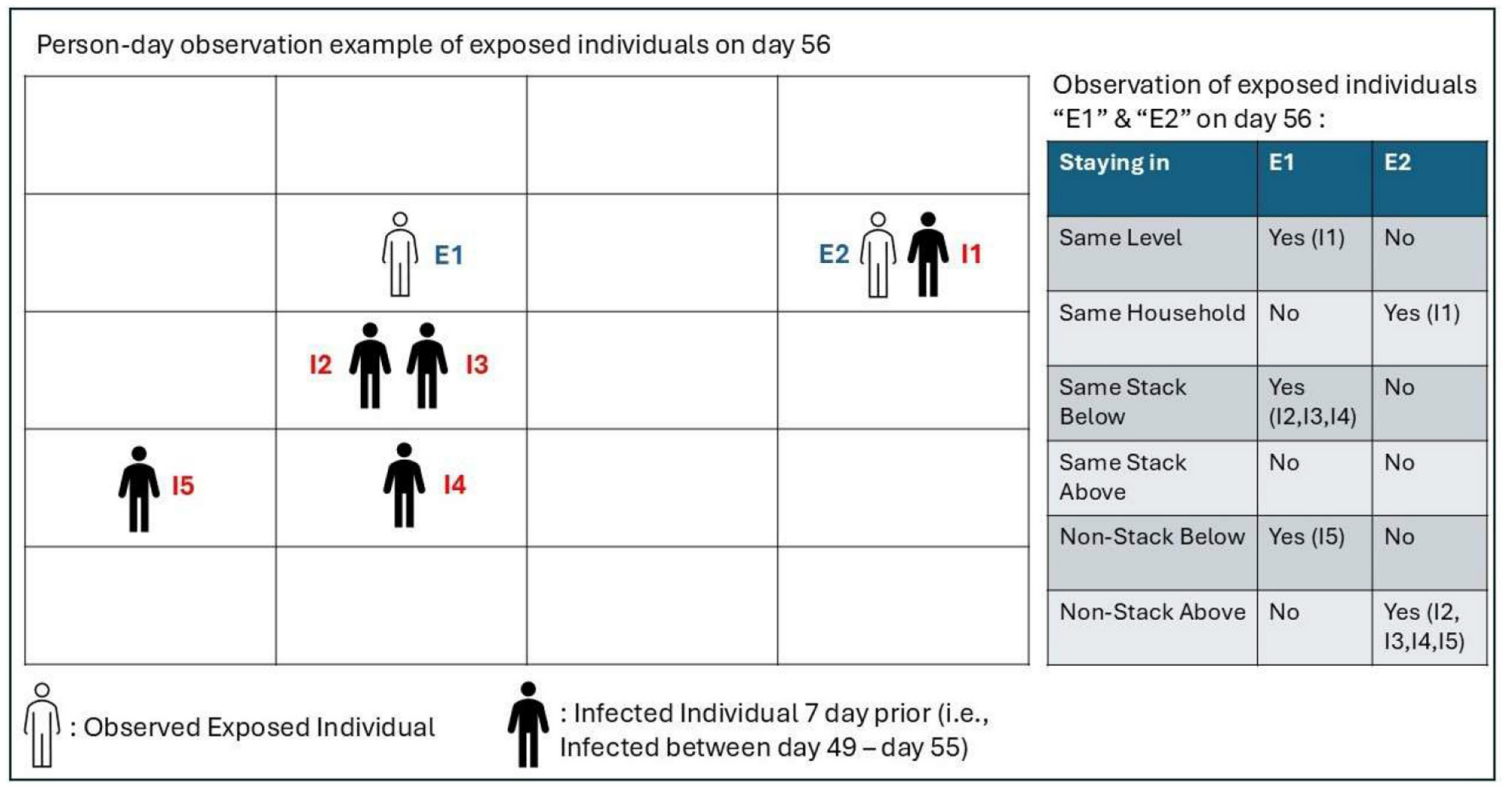
Illustration of how the location of exposed individuals (E1, E2) relative to potential index cases (I1–I5) living in the same HRA were factored in the logistic regression. The individuals' relative position in the grid is a simplified representation of their relative households' spatial position within the HRA. For example, E1 and I1 are from different households but on the same level (hence the “No” entry for “Same Household” and “Yes” entry for “Same Level” in the summary on the right). However, E2 is in the same household as I1, and there is no other potential index case on the same level (hence the “Yes” entry for “Same Household” and “No” entry for “Same Level” in the summary on the right). Similarly, I2, I3, and I4 are in households from the same vertical stack as E1 but on lower floors (hence the “Yes” entry in the third row “Same Stack Below” in the summary on the right).

For illustration, in Figure 2, “E1” and “E2” are observed exposed individuals on day 56, having been exposed to individuals I1, I2, I3, I4, and I5 who had been infected between day 49 and day 55 (7 days prior). The location of exposed individuals from index cases living in the same HRA were used as factors in the logistic regression as seen in the example from Figure 2. These exposed individuals will be classified as infected via HRA transmission if they test positive on day 56. Exponentiated coefficients from the logistic regression were derived, and the Odds Ratio (OR) was calculated, with higher OR values indicating a higher risk of SARS-CoV-2 infection of a potentially vulnerable individual. Simplistically, the OR quantifies the odds of an infection occurring among the individuals in one group (e.g., individuals living in the same vertical stack as an index case) to the odds of an infection occurring among the individual in another group (e.g., individuals living in a different vertical stack as an index case). Data analysis was carried out in STATA statistical software (StataCorp LP, version 17).

### Computational fluid dynamics model

**Figure 3:**
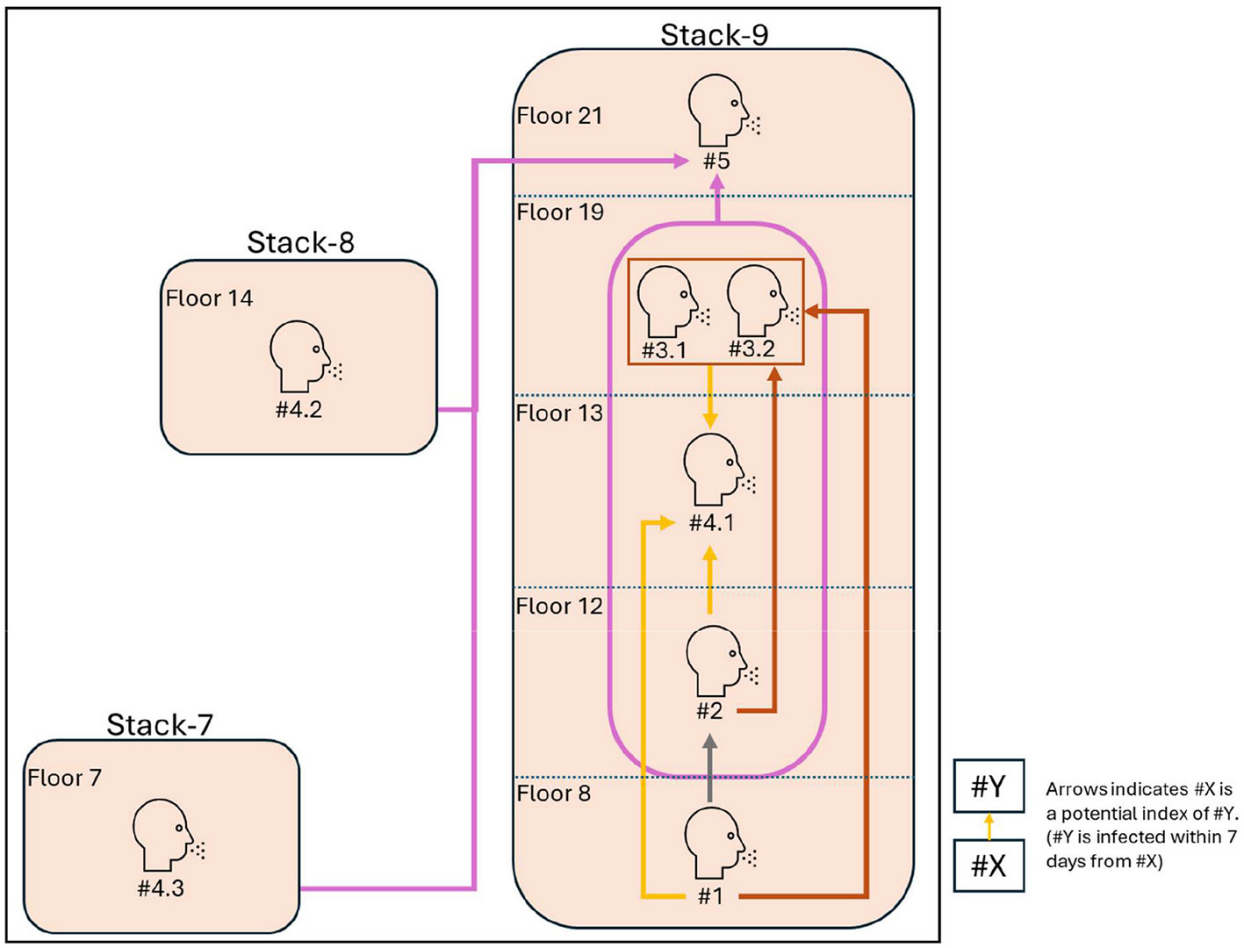
Vertical transmission patterns observed in the HRA selected for computational modeling, as assessed by infections diagnosed across unique days. Stack-7, Stack-8, and Stack-9 refer to 3 adjacent vertical stacks in the HRA (7th, 8th, and 9th units from left to right). The first number denotes the unique day on which the case was diagnosed, and the second number denotes the unique case(s) if there were more than one infection diagnosed on that day. The different-colored lines represent the potential index cases for each unique day that a case was diagnosed. For example, the gray arrow indicates that #2 could have been infected by #1, whereas the brown arrows indicate that #3.1 and #3.2 could have been infected by either or both of #1 and #2, and the yellow arrows indicate that #4.1 could have been infected by #1, #2, #3.1, or #3.2.

Out of the 18 HRAs included in the epidemiologic study, a single HRA with the highest number of unique unit transmissions across a singular vertical stack was chosen for further investigation on possible mechanisms for stack transmission via a computational model. Transmission in the HRA selected for computational modeling is illustrated in Figure 3. For simplicity, Figure 3 focuses on the single vertical stack that had the highest number of infections and the units that had infections of the same generation. Each arrow in the figure indicates a possible transmission, in which we define the potential index case as any case existing in the 7 days before a new infection.

### Digital model creation

A site survey of the location was conducted to facilitate the reconstruction of the HRA of interest and to verify its correspondence to information from OpenStreetMap. Reconstruction was subsequently done in accordance with simulation guidelines stipulated in Singapore's Building Construction Authority's Green Mark methodology, which was in turn based on prior literature describing best practices for the use of CFD modeling in urban studies (35–38). The horizontal extents of the explicitly reconstructed geometries are approximately 1 km × 1 km, and the building heights vary from 3 to 131.6 m. Original floor plans for each unit in the HRA were also used to reconstruct the exact layout of each unit.

**Figure 4:**
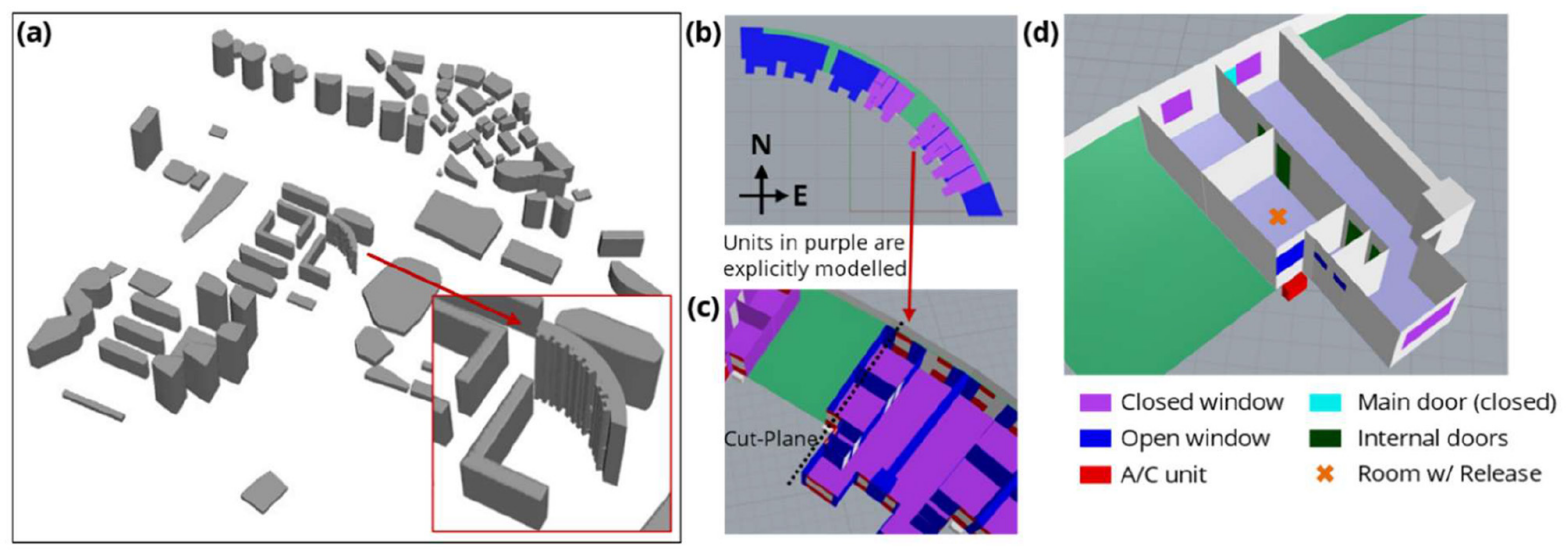
Schematics of **(a)** buildings around the HRA of interest; **(b)** the modeled units on every floor in the HRA of interest along a horizontal cut-plane; **(c)** the single unit corresponding to Stack-9 in the HRA of interest (9th unit going from left to right) along a horizontal cut-plane; **(d)** unit layout with the positions of all doors and windows.

Representative schematics of the studied HRA are provided in Figure 4. Explicit reconstruction of the surrounding buildings up to 500 m away from the HRA of interest was included to factor in the effect of the local built environment on wind flow surrounding the HRA, as depicted in Figure 4a. The explicitly represented units, including their relative positions, are depicted in Figures 4b, c. Six units surrounding the core unit of interest (referred to hereafter as Stack-9, as it is the ninth unit going from left to right) within the HRA were also modeled explicitly for all 24 floors of the HRA to facilitate numerical simulation of the trajectories of potential aerosolized viral particles entering and exiting individual units. Air-conditioning units and external fixtures (e.g., parapet walls) which may affect wind movements were also included. A full schematic of each unit's layout is presented in Figure 4d, illustrating all potential aerosol ingress and egress points (doors and windows). As is common for HRAs in Singapore, units primarily rely on natural ventilation, and there are no centralized, shared HVAC systems installed in this modeled HRA.

### Numerical implementation

All meshing and numerical simulations for this study were performed using the CFD software (ANSYS FLUENT version 21.2), using a similar methodology to that described in previous research by Ge et al. (30), where CFD simulation results were compared to experimental tests conducted with SF_6_ as the simulated aerosol. The computational domain was meshed using the Poly-Hexcore methodology which fills the bulk volume with octree hexes, a high-quality, layered poly-prism mesh in the boundary layer and conformally connects the two meshes with general polyhedral elements. A global mesh size of 0.025–40 m was applied, with local refinements at key features: 0.025 m for the source-emission box, 0.2 m for room surfaces, 0.1 m for air-conditioning outlets and inlets, 0.05–0.1 m for window openings, 1 m for the outdoor ground, and 5 m for surrounding buildings. A final mesh cell count of approximately 15 million was obtained. The Semi-Implicit Method for Pressure Linked Equations (SIMPLE) scheme was used to solve the fluid equations. Standard discretization schemes were applied for pressure, with second order upwind schemes being used for momentum, turbulent kinetic energy, turbulent dissipation rate and energy. Full buoyancy effects were also included. A scaled residual value of 10^−5^ was used as the convergence criterion for the RANS simulations and 10^−8^ for the passive scalar simulations.

The fluid phase is governed by the Navier–Stokes equation for conservation of mass and momentum and the energy equation:


$$\begin{array}{l} \nabla \cdot u=0 \end{array}$$ (1)

$$\begin{array}{l} \frac{\partial }{\partial t}u+\nabla \cdot \left(uu\right)=\nu_{eff}\nabla^{2}u-\nabla \frac{p}{\rho_{0}}\\ \;+\;\left[1-\beta \left(T-T_{0}\right)\right]g+S_{u} \end{array}$$ (2)

$$\begin{array}{l} \frac{\partial T}{\partial t}+\nabla \cdot \left(uT\right)=\nabla \cdot \left[\alpha_{eff}\nabla T\right]-\nabla \cdot q_{r}+S_{T} \end{array}$$ (3)

Where *u*, *p*, *T*, and *t* denote the air velocity, static pressure, static temperature, and time, respectively; *g*, *p*_0_, and *T*_0_ represent gravitational acceleration, reference density, and reference temperature; β, *v*_*eff*_, and α_*eff*_ are the thermal expansion coefficient, effective turbulence viscosity, and effective thermal diffusivity; *S*_*u*_ and *S*_*T*_ are the source terms for respective equations. RANS (Reynolds-Averaged Navier Stokes) simulations (specifically the realizable k-ε model) have been used in the study of urban environments and pollutants' dispersion as a good balance between computational cost and accuracy (39, 40). The realizable k-ε governing equation is as follows:


$$\begin{array}{l} \frac{\partial \left(\rho k\right)}{\partial t}+\nabla \cdot \left(\rho ku\right)=\nabla \cdot \left[\left(\mu +\frac{\mu_{t}}{\sigma_{k}}\right)\nabla k\right]\\ \;+\;G_{k}+G_{b}-\rho \varepsilon -Y_{M}+S_{k} \end{array}$$ (4)

$$\begin{array}{l} \frac{\partial \left(\rho \varepsilon \right)}{\partial t}+\nabla \cdot \left(\rho \varepsilon u\right)=\nabla \cdot \left[\left(\mu +\frac{\mu_{t}}{\sigma_{\varepsilon }}\right)\nabla \varepsilon \right]\\ \;+\;\rho C_{1}S_{\varepsilon }-\rho C_{2}\frac{\varepsilon^{2}}{k+\sqrt{v\varepsilon }}\;+\;C_{1\varepsilon }\frac{\varepsilon }{k}C_{3\varepsilon }G_{b}+S_{\varepsilon } \end{array}$$ (5)

Where *k* and ε represent the modeled turbulent kinetic energy and turbulent kinetic energy dissipation rate; ρ is the air density; σ_*k*_ and σ_ε_ are the respective turbulent Prandtl numbers; *G*_*k*_ and *G*_*b*_ represent the production of turbulent kinetic energy due to mean velocity gradients and buoyancy, respectively; *Y*_*M*_ represent the contributions from fluctuations to the overall dissipation rate; *C*_1_, *C*_2_, *C*_1ε_ and *C*_3ε_ are empirical constants; *S*_*k*_ and *S*_ε_ are the source terms for respective equations.

Furthermore, the passive scalar model in FLUENT is used to study the potential aerosol transmission. For this study, the steady-state flow field was calculated before any subsequent passive scalar simulations. The governing equation is defined as:


$$\begin{array}{l} \nabla \cdot \left(uc\;-D\;\nabla \cdot c\right)=S_{\text{c}} \end{array}$$ (6)

Where *c* denotes the passive scalar concentration; *D* represents the nominal diffusivity; *S*_*c*_ represents the passive scalar source term.

### Boundary conditions

Atmospheric boundary layer profiles for the mean wind speed, turbulent kinetic energy, and turbulence dissipation rate are prescribed at the inlet boundary based on prior literature (57).


$$U\left(z\right)=\frac{u*}{\kappa }ln\left(\frac{z+z_{0}}{z_{0}}\right)$$ (7)

$$k\left(z\right)=\frac{u{*}^{2}}{\sqrt{C_{\mu }}}$$ (8)

$$\varepsilon \left(z\right)=\frac{u{*}^{3}}{\kappa \left(z+z_{0}\right)}$$ (9)

Where u^*^ is the atmospheric boundary layer friction velocity derived using a reference mean wind speed at a specified reference height, and the aerodynamic roughness length *z*_0_; *k* is the von Karman constant; *C*_μ_ is a model constant defined as 0.09. The Davenport-Wieringa roughness classification was used to determine the appropriate aerodynamic roughness to reflect the degree of urban build-up (41).

A zero-pressure boundary condition is imposed on the outlet, while a zero gradient boundary condition is imposed on the other domain boundaries for the flow and passive scalar. Zero velocity is specified on wall surfaces. This model does not explicitly handle aerosol deposition on surfaces, and any potential fomite transmission. For comparison across scenarios, a normalized source is introduced into the passive scalar simulation at a consistent location near the window in the bedroom of interest (where the putative index case is located).

**Figure 5:**
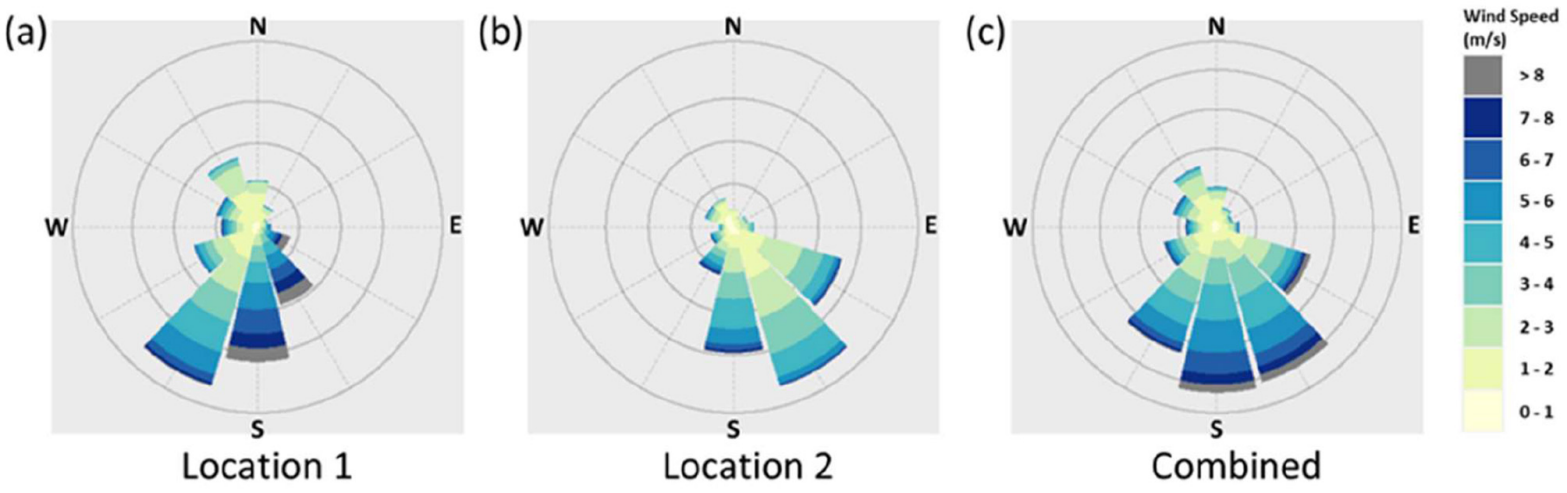
Wind rose of the prevailing winds (with the four cardinal directions indicated) recorded at **(a)** location 1 which is a common reference station in Singapore; **(b)** location 2 which is the closest station to the HRA of interest; and **(c)** composite of Locations 1 and 2. The radial extent of each wedge corresponds to the frequency of wind occurrences from that direction (with concentric circles marking increasing counts), while the color within each wedge indicates the distribution of wind speeds as per the scale bar on the right. For consistency, the time period analyzed for the wind rose (and subsequent CFD simulation) corresponds to the period under analysis in the epidemiology study.

To determine the wind direction, open-source meteorological information for the period corresponding to the epidemiological dataset was analyzed. The period between June and September corresponds to the southwest monsoon season in Singapore and is associated with southerly and southeasterly prevailing wind directions (42). Historical records indicate a mean velocity of 2.8 and 2.0 m/s at a 15 m reference height for South and North prevailing winds, respectively (33). Meteorological data from June 2021 verified the consistency of prevailing wind conditions with these historical observations. Records from a standard reference meteorological station near the eastern region of Singapore and another meteorological station on mainland Singapore closest to the HRA of interest were assessed, and a composite of observed prevailing winds over these two locations was generated. The dominant wind direction was assessed to be the southerly wind direction, in line with prior historical records, and this was selected for numerical simulation. The wind rose of the prevailing winds from the reference stations and the composite are illustrated in Figure 5.

The numerical simulations were conducted under two permutations. In the first variant, which used a southern prevailing wind, the ingress and egress locations within the units were modified according to two configurations, “mostly-open” and “mostly-close.” The worst-case scenario, a “mostly-close” scenario, was considered the baseline, in which the main door and all internal doors are assumed to be closed, and all windows are closed except for the window from the bedroom with a simulated release, and the accompanying bathroom windows (which are typically left open). The “mostly-open” scenario had all the windows and doors (besides the main door) modeled as open. The specifics of each configuration are expanded in Table 1. In a second set of experiments, the inlet boundary condition was modified to the direct opposite direction (Northerly direction) to investigate the potential impact of prevailing winds. Hence, these two permutations were assumed to represent the two most extreme scenarios (with maximal and minimal ventilation, respectively).

**Table 1:** Residential unit configuration permutations.

**Ingress/Egress location**	**“Mostly-open” configuration**	**“Mostly-close” configuration**
Unit main door	Closed	Closed
Unit internal doors	Open	Closed
Unit bedroom 1 window	Open	Closed
Unit bedroom 2 window (corresponding to the room with release in Figure 4d)	Open	Open
Unit living room windows	Open	Closed
Unit bathroom windows	Open	Open

## Results

### Epidemiology

Based on the collected dataset, logistic regression was used to estimate the odds of SARS-CoV-2 infection within 7 days among potentially vulnerable individuals living in the same household, level, and vertical stack as an index case. The regression controlled for vaccination status, age group, gender, race, and residency status as covariates. As individuals can be concurrently exposed to multiple potential index cases within their 7-day exposure window, the logistic regression model includes all potential exposures simultaneously as covariates, allowing for estimation of the adjusted odds ratio for each exposure type while controlling for the presence of all other concurrent exposures.

Results showed that residents were at increased risk of SARS-CoV-2 infection within 7 days if they were living on the same level (OR 1.98, 95% CI 1.44–2.72), same stack (OR 2.03, 95% CI 1.41–2.93), or same household (OR 89.41, 95% CI 62.18–128.57) as an index case (Table 2). Consistent with previous reports, the risk of infection from another household member was much higher than any extra-household infection risk (43). Based on the epidemiology, the elevated risk of infection for residents on the same level, same stack, or same household was all found to be statistically significant (*p*-value < 0.05). Critically, to the best of our knowledge, this is the first quantification of the spatial dependence of infection risk across HRAs, albeit limited to HRAs of the type present in Singapore.

**Table 2:** Infection risk within the HRA.

	**Person-days (*n*)**	**Infected (*n*)**	**Infected (%) ($\frac{n_{infected}}{n_{person-days}}$)**	**Infected OR**	***p*-Value**
**1 May**−**20 Sep 2021**					
**Staying at the same level as the index case**					
No	224,160	149	0.07%	1.00 (.-.)	–
Yes	35,612	62	0.17%	1.98 (1.44–2.72)	2.35E-05
**Staying in the same stack as the index case**					
No	227,857	140	0.06%	1.00 (.-.)	–
Yes	31,915	71	0.22%	2.03 (1.41–2.93)	1.41E-04
**Staying in the same household as index case**					
No	257,367	119	0.05%	1.000 (.-.)	–
Yes	2,405	92	3.83%	89.41 (62.18–128.57)	7.61E-130
Total	259,772	211	0.08%		

To further isolate risks associated with transmission along the same stack, 92 cases with household exposure in the preceding 7 days were excluded from the subsequent regression analyses. Residents living above an index case had a higher risk of infection within 7 days (OR 7.42, 95% CI 4.91–11.21) compared to residents living below the index case (OR 2.56, 95% CI 1.54–4.25). Further analysis by proximity showed that the risk of infection decreased with increasing distance from the index case, i.e., the number of floors from the index case. Residents living within 1–2 levels above an index case had a higher risk of infection within 7 days (OR 6.32, 95% CI 3.55–11.27) than residents living three or more levels above an infected case (OR 3.72, 95% CI 2.11–6.56; Table 3). Critically, residents living in units above or below the index case but on a different stack had higher risks of infection, which were significantly less statistically significant (*p*-value >0.05) than the other scenarios where the residents were in the same stack (*p*-value < 0.05).

**Table 3:** Infection risk within the HRA by proximity.

	**Person-days (*n*)**	**Infected (*n*)**	**Infected (%) ($\frac{n_{infected}}{n_{person-days}}$)**	**Infected OR**	***p*-value**
**1 May**−**20 Sep 2021**					
**Staying in the same stack below the index case**					
No	240,282	100	0.04%	1.000 (.-.)	–
Yes	17,085	19	0.11%	2.56 (1.54–4.25)	2.94E-04
**Below (Within 1 or 2 levels)**					
No	251,962	104	0.04%	1.00 (.-.)	–
Yes	5,405	15	0.28%	4.36 (2.35–8.08)	3.02E-06
**Below (3 or more levels)**					
No	244,839	108	0.04%	1.00 (.-.)	–
Yes	12,528	11	0.09%	1.41 (0.75–2.66)	0.29
**Staying in the same stack above the index case**					
No	242,211	81	0.03%	1.000 (.-.)	–
Yes	15,156	38	0.25%	7.42 (4.91–11.21)	1.61E-21
**Above (Within 1 or 2 levels)**					
No	251,791	99	0.04%	1.00 (.-.)	–
Yes	5,576	20	0.36%	6.32 (3.55–11.27)	3.96E-10
**Above (3 or more levels)**					
No	246,912	98	0.04%	1.00 (.-.)	–
Yes	10,455	21	0.20%	3.72 (2.11–6.56)	5.81E-06
**Staying below but not the same stack as the index case**					
No	32,989	15	0.05%	1.000 (.-.)	–
Yes	224,378	104	0.05%	1.50 (0.86–2.60)	0.15
**Staying above but not the same stack as the index case**					
No	134,918	52	0.04%	1.000 (.-.)	–
Yes	122,449	67	0.05%	1.40 (0.95–2.04)	0.09
Total	257,367	119	0.05%		

### Computational fluid dynamics model

**Figure 6:**
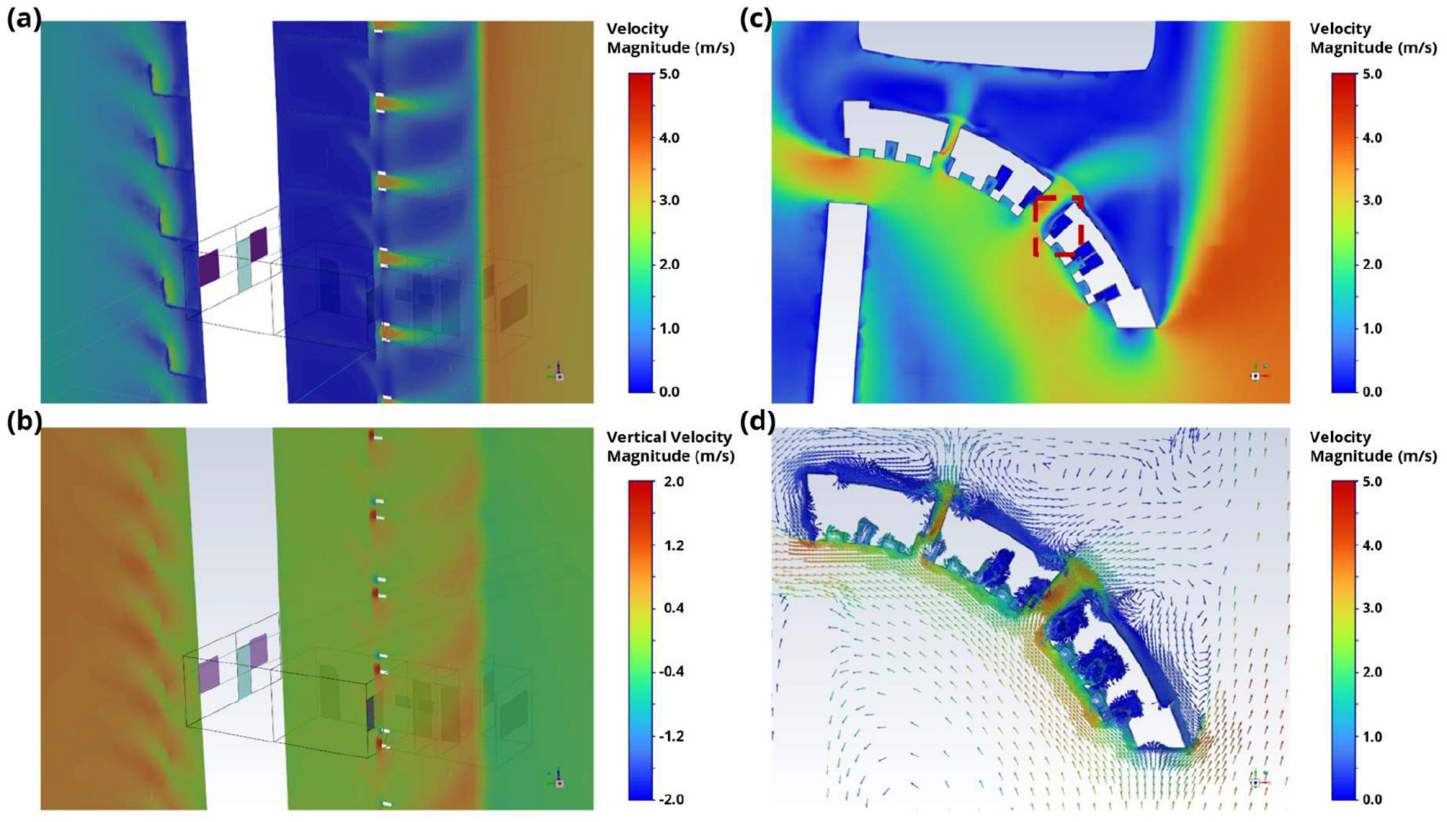
**(a)** Contour of velocity magnitude along a vertical cut-plane through the bedrooms in Stack-9, where a potentially infected individual might be. **(b)** Contour of vertical z-velocity magnitude along a vertical cut-plane through the bedrooms in Stack-9, where a potentially infected individual might be. **(c)** Contour and **(d)** vectors of velocity magnitude along a horizontal cut-plane through the HRA of interest for a mid-height floor (Floor 13 out of 24 floors). A red box is drawn around the bedroom in Stack-9 where a potentially infected individual might be in **(c)**.

Based on the model described in Figure 4, velocity contour and vector plots from CFD for the southerly wind scenario are presented in Figure 6 to illustrate the characteristics of the airflow and ventilation around the studied HRA. Notably, the southerly wind scenario is of particular interest for this HRA, as it was the predominant wind direction (due to the prevailing monsoon in Singapore) when the cluster occurred (as shown in Figure 5). The location of the vertical cut-plane depicted in Figures 6a, b corresponds to the dotted line in Figure 4c. Outlines of a single unit are also included in the contour plots to facilitate visualization. Total velocity magnitude and vertical velocity magnitude contours in Figures 6a, b indicate that the airflow around the bedroom window in Stack-9 is very low based on the southerly wind scenario. The reason for this is illustrated in Figures 6c, d, where the velocity contour and velocity vectors in the horizontal plane show how the area along the building façade near the bedroom window is shielded from the main path of the oncoming wind. This causes the wind velocity near the bedroom window to be significantly lower than in the surrounding area. Given the relatively low velocities around the bedroom, the slight vertical velocities observed in Figure 6b are relatively more dominant, and this can cause an upward bias in the movement of aerosols originating from units in this stack.

**Figure 7:**
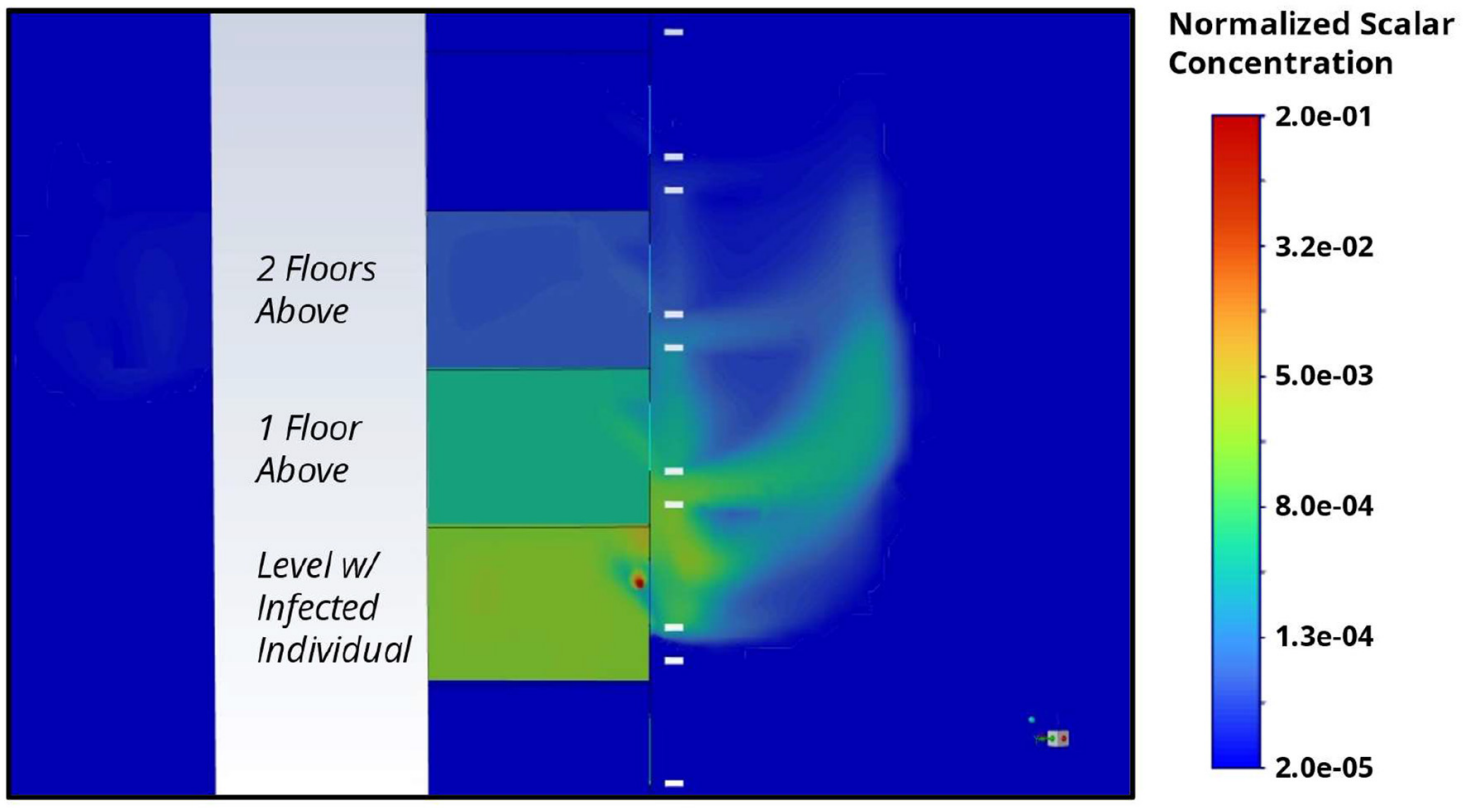
Contour of normalized passive scalar concentration along a vertical cut-plane through Stack-9 in the HRA of interest. The passive scalar is assumed to originate from a random floor in the stack (representing a potentially infectious individual recuperating in the bedroom), and the steady-state distribution is presented.

The constructed numerical model can also be used to simulate the release of a passive scalar source, corresponding to potential aerosolized viral particles being released from an infected individual in the unit's bedroom. This can be used to study the potential movement of aerosolized viral particles from an infected person in a single unit to units along the HRA. It should be noted that large droplets are unlikely to cause airborne transmission across the vertical stack. Hence, they are not modeled by the passive scalar methodology. Contours of the passive scalar concentrations (normalized to the release concentration) are plotted for a vertical cut-plane across the stack of interest in Figure 7. The normalized passive scalar concentration closely resembles the vertical velocity profile presented in Figure 6b as the aerosols are being transported by the local airflow. Released particles were shown to exhibit a vertical bias across the stack from a putative infected individual in a unit to the units directly above it. Furthermore, consistent with the observed epidemiology, there is a strong dependence on proximity, with the nearer units (1–2 floors above) exhibiting elevated levels of the potential aerosol.

To ensure consistency in the patterns observed, a random subset of seven floors (approximately half of the units between Levels 4 and 19) was chosen for simulation, and the average normalized passive scalar concentration observed for 1–2 floors above, and for three or more floors above, was collated. The simulation results are then used to compare the concentration of potential aerosolized viral particles in units 1 or 2 levels above the release unit to that for units three or more levels above, as per the epidemiology presented in Table 3.

**Figure 8:**
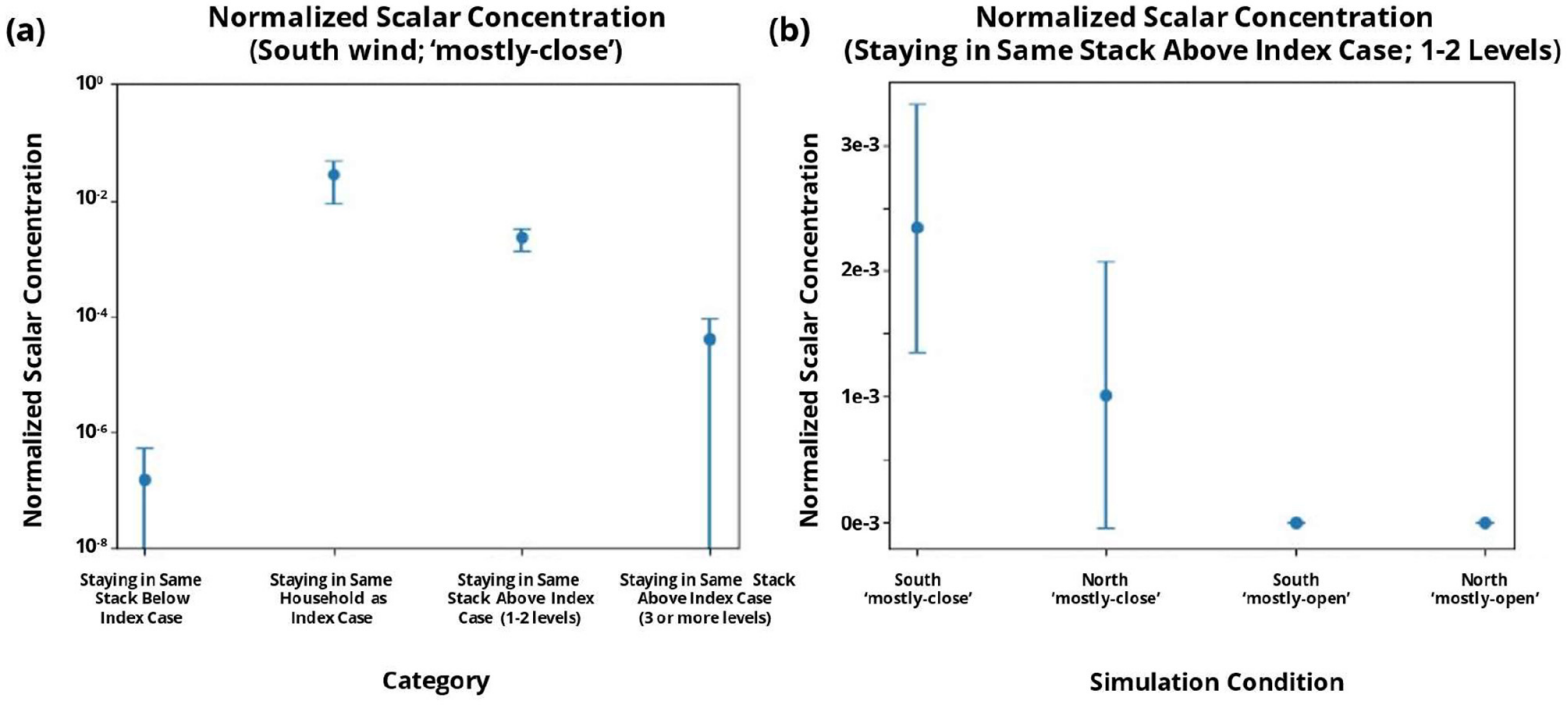
Error-bar plots (with mean and standard deviation) of the passive scalar concentration normalized to the emission concentration for the simulated units in Stack-9. Plot **(a)** presents the distribution of normalized concentrations for different relative positions of the index case location, and **(b)** presents the distributions under different simulation conditions for the scenario where the putative index case is located within the 2 levels below.

It was observed that the simulated aerosols could reach the higher units at significant concentrations along Stack-9 for the southerly wind direction, as illustrated in Figure 8a. Aligned with epidemiological findings, the concentration in the same household is the highest, although it should be noted that transmission in the same household is likely to involve a combination of both airborne, contact, and fomite-based transmission. Furthermore, the concentration is orders of magnitude lower in both scenarios: when an individual stays below the index case or three or more levels above it. Hence, the airflow simulations provide a good qualitative match to the epidemiological observation that there can be an increased risk of vertical stack transmission when staying within 1–2 levels above an index case for this particular combination of prevailing winds and unit configuration.

A simulation of particles released from Stack-9 based on a northern prevailing wind was also conducted (the exact opposite of the predominant southerly wind that was present during the outbreak of this cluster). The extent of aerosol transmission in the vertical stack is reduced when the prevailing wind is coming from the north, as presented in Figure 8b. Empirically, the clusters observed also occurred during the Southwest monsoon season when southerly winds are dominant. This clearly shows that the potential for vertical stack transmission can be significantly affected by the complex interplay between the neighboring local built environment and prevailing wind direction.

Furthermore, an internal configuration with enhanced cross-ventilation across the unit (“mostly-open”) was explored, whereby all the windows and doors (besides the main door) were modeled as open. In this scenario, there was virtually no transmission along Stack-9, with a greatly alleviated risk of transmission relative to the “all-close” scenario for both North and South prevailing winds. This shows the importance of keeping the unit well-ventilated to avoid low horizontal velocities around the windows of a potentially infected unit. This can help mitigate the potential upward spread of aerosols from a unit with an infected individual, and is also consistent with prior recommendations to maximize natural ventilation in other settings (44).

**Figure 9:**
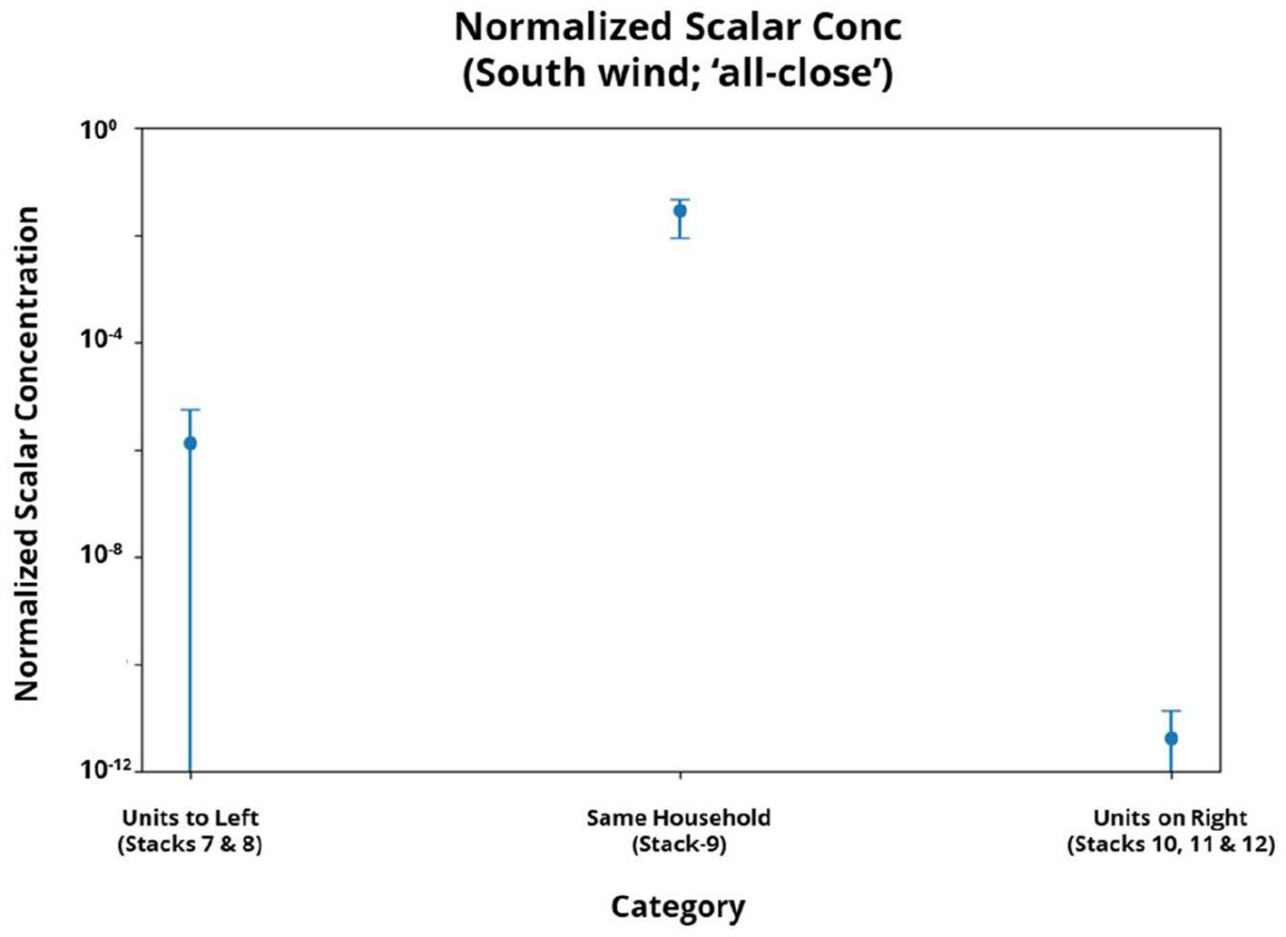
Error-bar plot (mean and standard deviation) of the passive scalar concentration normalized to the emission concentration for the units to the left and right of Stack-9.

As the epidemiology revealed an increased OR for cases within the same level as the index case, the average normalized passive scalar concentration for the units to the left and right of the Stack-9 units were also analyzed, as presented in Figure 9. The observed passive scalar concentrations are qualitatively consistent with the velocity magnitude and velocity vector contours presented in Figure 6, where the units in Stacks 10, 11, and 12 are upwind of Stack-9 and are therefore much lower in concentration than Stacks 7 through 9. Nonetheless, the obtained passive scalar concentrations in the units to the left and right of the unit containing the putative infected individual (Stack-9) remain orders of magnitude lower than the observed concentrations in the units in the same stack above the index unit (as per Figure 8). The extremely low concentrations suggest that aerosol transmission via the external façade of the HRA is unlikely to cause infection for individuals on the same level.

## Discussion

The epidemiological analyses suggest an elevated risk of infection across multiple scenarios with significant statistical significance. Nonetheless, the cases identified were not linked via genomic sequencing or air samples, which would have provided more robust evidence of transmission. Cases may still have had exposure to multiple infected cases in the same level or stack, which poses a challenge in accurately identifying the source of infection. We acknowledge this as a study limitation inherent to the operational context in which the outbreak investigations were conducted, rather than a methodological shortcoming of the analytic approach. Future studies could incorporate comprehensive environmental sampling and genomic sequencing or homology analyses of viral isolates from both confirmed and suspected cases to validate epidemiologic linkages. Such integration of molecular data with spatial and modeling analyses would strengthen causal inference and clarify whether observed transmission patterns represent true intra-building spread. The use of a person-day observation model also poses a limitation, as individuals are removed once infected. Whilst this prevents potential erroneous counts of infection in cases of prolonged SARS-CoV-2 infection, it precludes the ability to factor in reinfection in individuals, which is a known possibility. This limitation is noted, but it does not materially affect the primary interpretation of spatial risk gradients within HRAs during the study period.

Furthermore, HRAs are a highly complex setting which enable a variety of modes of transmission. Whilst there were prevailing social measures curbing the movement of residents and limiting their movements beyond their homes, there were no specific stipulated measures limiting the movement of residents outside of their apartments, and along the corridors and common areas of the blocks. Hence, contact-based transmission remains a plausible route. Residents living on the same level as an infected case would likely have been exposed to common high-touch areas, such as the lift lobbies and stairwells, especially in light of prior reports on possible infection through fomite transmission (45). SARS-CoV-2 has been observed to be viable and infectious on surfaces for up to days, depending on the levels shed, making fomite transmission a possibility (46). These possible points of exposure are potentially reflected in the increased risk of SARS-CoV-2 infection among persons living on the same level as infected cases.

Earlier studies have shown that SARS-CoV-2 is able to transmit viral particles in the form of aerosols (4). Similarly, an increased risk of infection among residents living above an index SARS-CoV-2 case on the same stack was observed in the epidemiology, especially for residents living within two floors above an infected unit, suggesting potential directionally biased transmission of aerosols upwards. The impact of physical proximity further suggests a physical route for transmission (in addition to other possibilities such as transmission through shared facilities). These findings are prima facie consistent with prior observations in which SARS-CoV-2 cases had been recorded in the same vertical stack of an HRA with no other epidemiological links.

In previous studies, it was postulated that potential spread occurred via drainage or structural elements in the bathroom. In their study, Hwang et al. investigated possible aerosolized viral particle transmission through the sewer stack and vertical air shaft in the core of the HRA (22). Whilst Hwang et al. postulated potential transmission through the air shaft, the HRAs under study in this study do not have a similar vertical air shaft, necessitating the need to consider other potential routes of transmission (22). Separately, others have hypothesized that virus-laden aerosols may be generated from hydraulic interactions between the flushing of waste and the surrounding stack pipes (45, 56), and transmitted vertically through a combination of depleted U-traps and potential chimney effects. However, these prior reports also suggested that higher floors are particularly vulnerable when the chimney effect is accentuated with greater temperature differences and low humidity, with multiple reports suggesting that this could also be a reason for the majority of stack transmission observed during the winter months (25, 45, 47). Conversely, the results indicate that the risk of infection decreases when the individual is three or more floors above the index case, regardless of the height of the unit of the index case. Furthermore, the humidity in Singapore is high year-round, which may also reduce the possibility of the drainage drying out and transmission through the drainage stacks. Hence, the epidemiology observed in this study, which was suggestive of directionally-biased spread (more elevated for floors above the infected units), was not fully consistent with previously proposed mechanisms of transmission, motivating a consideration of alternative causes of aerosolized viral particle transmission, including via the external façade of the HRA.

To evaluate this possibility, a CFD model was developed to acquire insight into airflow dynamics around the HRA and assess the potential for airborne transmission of aerosolized viral particles within a vertical stack. The low velocities observed along Stack-9 allow for the upward transport of potential aerosols from the bedroom windows, and ingress of these potentially infectious aerosols into other units along the stack. In conjunction with the “mostly-close” scenario wherein most ingress and egress points are closed and cross-ventilation through the unit is obstructed, this leads to potential ventilation patterns which will increase concentrations in units along the stack. This is clearly shown in Figure 8, where aerosols released under specific conditions (i.e., southerly wind and a mostly closed configuration) resulted in elevated concentrations of aerosols in the units above the infected unit. Notably, the downward movement of particles is limited and falls off significantly. This qualitatively aligns with the epidemiological finding that the risk of infection is higher for an individual living in a unit above the index case, compared to an individual living in a unit below the index case, further highlighting the value of a joint investigation from both the epidemiological and numerical modeling perspectives.

Dose–response modeling studies have established that infection probability increases with cumulative inhaled viral dose (12, 49, 58). By anchoring our CFD-simulated concentration maps in this dose–response relationship, this reinforces the possibility that observed aerosol accumulation, particularly in vertically adjacent units, may lead to cumulative inhaled doses exceeding infectious thresholds.

Whilst the epidemiologic investigations and modeling study support the mechanism of aerosolized viral particle transmission via the external façade of the HRA, transmission is complex with multiple points of interaction, and other mechanisms of transmission remain unexplored. For example, although the epidemiology suggested a high OR for individuals living on the same level as an infected index case, we note that the airflow model did not suggest elevated concentrations in the neighboring units on the same level. This divergence suggests that the increased risk among individuals on the same level may encompass other mechanisms, such as fomite transmission or some social interactions. We also note that prior reports of airflow-mediated cross-corridor transmission have been for instances of double-loaded corridors, where the airflow is correspondingly poorer, which is not the case for this particular HRA (50).

The results from the airflow model based on a single selected HRA may not reflect airflow patterns across all HRAs, but rather demonstrate the feasibility of viral aerosol transmission vertically along a building stack in a particular scenario in which infection has been observed. Nonetheless, as part of Singapore's public housing, the 18 HRAs do exhibit some architectural consistency, especially in their corridor-based access, unit layouts, and the general absence of any centralized HVAC. Just as prior studies have suggested the importance of meteorological factors in facilitating transmission, the direction and strength of seasonal prevailing winds may also be worth further study (48). Moreover, the two scenarios studied (“mostly-open” and “mostly-close”) were chosen as representative of the two most extreme scenarios (minimal and maximal cross-ventilation through the unit). They may not fully represent the actual day-to-day situation. Fundamentally, the configuration of the windows and doors is a dynamic variable that depends on occupants' behavior with significant stochasticity. Future research to more thoroughly investigate the impact on airborne transmission when individual windows and doors are randomly opened or closed could yield additional insights. Furthermore, studies that model a broader range of HRA configurations would help characterize the variability of airflow patterns across different layouts and micro-environments. Complementary environmental sampling, including air and surface collections from units and common areas, could be incorporated to provide empirical validation for aerosol distribution patterns. Nonetheless, the reduced risk observed for the “mostly-open” scenarios (maximal cross-ventilation across the unit) is consistent with prior reports that improved ventilation practices can reduce the risk of infection in the residential setting (49).

This study, based on an empirical analysis of 18 HRAs with known clusters and airflow modeling of a specific HRA, provides evidence supporting the hypothesis that vertical stack transmission can occur in HRAs. However, it also highlights the need for future extensions to address how broadly this applies across all types of HRAs. Environmental sampling (e.g., from various locations in the HRA with known infections) and quantitation of circulating viral aerosols in future similar studies would have been invaluable for further validation of the mechanistic modeling. Further investigations and modeling studies across a variety of HRA configurations can unveil other potential vulnerabilities. In particular, we note that *in silico* reproduction with different simulation scenarios can have two possible outcomes, especially when analyzed in conjunction with epidemiologic associations, which are themselves inherently correlational. If they match, this provides insight into potential mechanistic routes of transmission; however, it should be noted that this is not, by itself, definitive without real-world validation, nor is it necessarily an exclusive or the most important cause. Conversely, discrepancies such as those observed for same-level transmission and simulated airflow patterns across different simulation scenarios in this study can also drive further investigation into other potential transmission mechanisms, including non-aerosol mechanisms such as corridor interactions or fomite exposure. Finally, it is important to note that observable particle concentrations need not constitute an absolute risk, and infection remains dependent on a multitude of factors. Transmission within the household and via other routes (e.g., fomites) remains a key driver of infection, necessitating distinct mitigation efforts.

## Conclusion

HRAs form the bulk of housing in many urban settings; Singapore itself has a significant population (over 80%) residing within HRAs (51). Moreover, in extenuating circumstances, especially in a land scarce setting like many urban metropolises, HRAs have been utilized as locations for quarantine; at the height of SARS-CoV-2 infections in Singapore, up to 40% of cases diagnosed were subject to home isolation, with home recovery being the default mode of care for fully vaccinated residents unless medically or socially contraindicated (52). A related study in Hong Kong, another city with significant HRAs, reported significantly increased risk of vertical transmission, although they did not conduct any accompanying computational study (53). In such settings, knowledge of potential risk and transmission routes is important to ensure that corresponding strategies for mitigation and prevention can be developed and implemented.

Against this backdrop, the findings of this study are particularly timely. This study provides the first epidemiology-based quantification of infection risk across different spatial configurations within HRAs, demonstrating that residents faced significantly elevated risk of SARS-CoV-2 infection within 7 days if they lived on the same level (OR 1.98), in the same vertical stack (OR 2.03), or in the same household (OR 89.41) as an index case. This provides compelling evidence that SARS-CoV-2 transmission within HRAs is not merely a theoretical concern but a quantifiable phenomenon.

Furthermore, CFD modeling reveals that airborne viral particles can travel along vertical building stacks under specific conditions, potentially explaining observed epidemiology. This complements prior literature focused on drainage stack transmission, instead highlighting external façade-mediated aerosol dispersion as a viable alternative mechanism for vertical spread. While the epidemiological results are based on multiple HRAs, the CFD simulation results are specific to a single HRA. They should be interpreted as mechanistic insights rather than as broadly generalizable infection probabilities. Ultimately, HRAs have significant variation in their internal and external configurations, all of which can potentially influence particle movement and transmission risk. The permutations studied in this research (prevailing wind and extent of cross-ventilation) already unveil significant heterogeneity in the resultant transmission observed, motivating more detailed studies across different scenarios to further elucidate aerosolized particle movement and the extent to which they can account for observed epidemiologic trends.

As cities continue to densify and infectious disease threats evolve, interdisciplinary research integrating epidemiology, fluid dynamics, and environmental science will be crucial in shaping resilient public health policies. Our study demonstrates how advanced modeling approaches can inform pandemic preparedness, ensuring that urban environments and policies are designed to mitigate, rather than exacerbate, the risk of airborne disease transmission. The body of knowledge from subsequent modeling efforts, as guided by observed epidemiology, may serve to advise future public health measures and requirements, even as insights from prior studies in other settings have already started to lead to recommendations and a re-thinking of the public space (54, 55).

## Data Availability

The datasets presented in this article are not readily available because, due to data governance policies and confidentiality considerations, the authors are unable to share individual-level datasets, as these contain sensitive information. However, the authors are able to provide aggregated, non-identifiable summary statistics derived from the datasets used in this study upon reasonable request. Requests to access the datasets should be directed to: samuel.chong@mohh.com.sg.
